# Reconstruction of Cheek Defects

**Published:** 2011-06-24

**Authors:** Joseph M. Meyerson, Alison McAnneny

**Affiliations:** Department of Plastic Surgery, The Ohio State University College of Medicine, Columbus, OH

## DESCRIPTION

A 46-year-old woman presents for excision of a lesion over her left cheek that has been increasing in size over the past year.

## QUESTIONS

1. **What is the differential diagnosis for malignant lesions of the cheek?**
  **What are the challenges of cheek reconstruction?**
  **What reconstructive options are used to repair cheek defects?**

## DISCUSSION

The differential diagnosis for malignant lesions of the cheek includes basal cell carcinoma, squamous cell carcinoma, and melanoma. These malignancies have multiple risk factors in common: excessive sun exposure, increased age, and low Fitzpatrick skin type. Each has a characteristic appearance, but many may have atypical presentations. Basal cell classically presents with rolled borders and pearly, translucent edges with associated telangiectasias. Squamous cell carcinoma often forms a central area of ulceration. Malignant melanoma is characterized by asymmetry, irregular borders, and variegated color. Other less common tumors may occur on the cheek, some of which include metastasis, frequently from adnexal tumors, neuroendocrine tumors such as Merkel cell carcinoma and growth from underlying structures as with exophytic parotid tumors. The differential is vast and must be considered when approaching a possible tumor of the cheek.

There are multiple challenges to the reconstruction of cheek defects, due to their visible location and limited supply of local tissue. The anatomy surrounding the cheek is highly involved with facial expression and includes the forehead, eye, nose, mouth, and ears. Once the overlying lesion has been removed reconstructive efforts must preserve important underlying structures, and when possible, restore facial aesthetics. Consideration of the lower eyelid is of particular importance during reconstruction of medial cheek defects. Excess tension may cause lower lid malposition, which can result in epiphora, lagophthalmos, and exposure keratitis.

Reconstructive options for cheek defects include primary closure, skin grafts, local or regional flaps, and free tissue transfer. The method of reconstruction is based on the defect's size and location, functional deficits, and structural involvement (ie, skin, muscle, nerves, bone). Restoration of function, preservation of facial symmetry, minimizing and camouflaging scars and contour irregularities are all inherent to reconstruction. In the presented case, a preauricular defect from a squamous cell carcinoma excision was closed with a rotation flap, which was designed to utilize excess cervical skin. Final scar shape, size, and placement must be considered when designing the method of closure. If possible, placing incisions within relaxed skin tension lines (eg, wrinkles), along natural contours of the face (eg, the nasolabial fold), or along the hairline will help camouflage scars and result in a more aesthetically pleasing result.

## Figures and Tables

**Figure:**
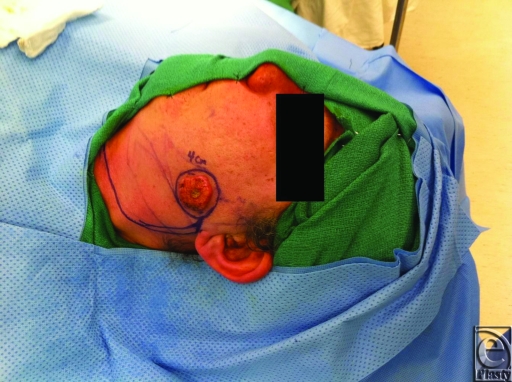


**Figure:**
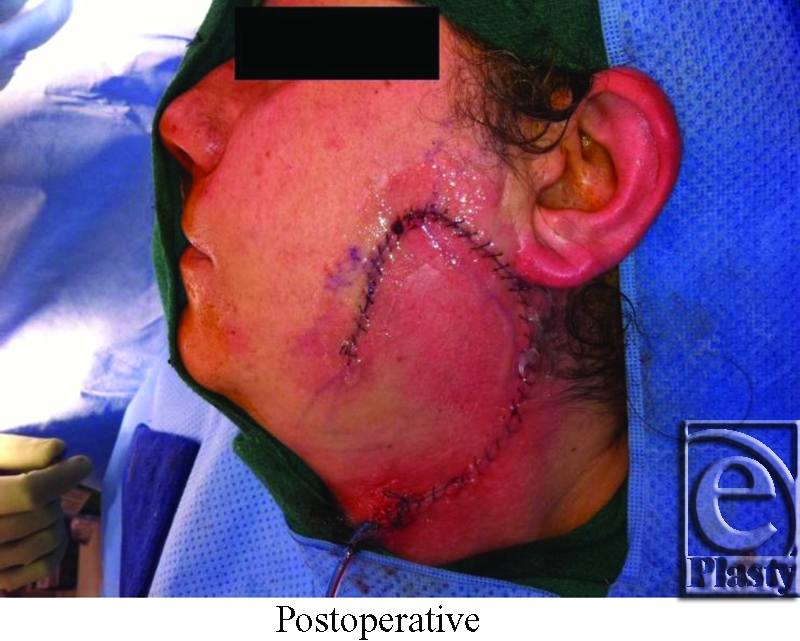


## References

[1] Jowett N, Mlynarek AM (2010). Reconstruction of cheek defects: a review of current techniques. Curr Opin Otolaryngol Head Neck Surg.

[2] Heller L, Cole P, Kaufman Y (2008). Cheek reconstruction: current concepts in managing facial soft tissue loss. Semin Plast Surg.

[3] Pletcher SD, Kim DW (2005). Current concepts in cheek reconstruction. Facial Plast Surg Clin N Am.

[4] Summers BK, Siegle RJ (1993). Facial cutaneous reconstructive surgery: facial flaps. J Am Acad Dermatol.

